# The importance of biobanking in molecular taxonomy, with proposed definitions for vouchers in a molecular context

**DOI:** 10.3897/zookeys.365.5875

**Published:** 2013-12-30

**Authors:** Jonas J. Astrin, Xin Zhou, Bernhard Misof

**Affiliations:** 1 Zoological Research Museum Alexander Koenig (ZFMK), Centre for Molecular Biodiversity Research, Bonn, Germany; 2 BGI, China National GeneBank, BGI-Shenzhen, Shenzhen, China 518083

DNA barcoding and molecular or integrative taxonomy projects are among the most valuable sources for biobank specimens of wild organisms, thanks to – among other aspects – the high level of specimen diversity and thanks to a thorough taxonomic coverage. Specimens used to build barcoding reference libraries tend to be accompanied by deeper and higher-quality data than samples from many other sources, as they are often contributed by taxonomists, and identifications are cross-checked through barcode analysis. Vouchering of morphological specimens in natural history collections is a prerequisite for proper barcoding, which is advantageous for biobanking as well, as biobank samples should always be linked to specimen vouchers. As a further added value, barcoding provides an inherent, molecular species ID tag to the processed biobank sample.

Banked barcoding samples can greatly catalyze taxonomy, as well as many other fields of application, such as the emerging large genome sequencing projects that are constantly increasing the demand for well-preserved samples from a multitude of different species (see Wong et al. 2012).

Considered from the opposite perspective of the synergy, barcoding can benefit greatly from biobanking as well. Biobanking enables the expansion of barcoding datasets with biobanked samples from other projects. It also offers the possibility to add new barcoding markers any time in the future, e.g. scaling up to ‘next-generation barcoding’ (e.g. Taylor and Harris 2012) if feasible (manageability of data, NGS and data handling cost, performance in mixed samples, etc.), without the necessity of repeating the time-consuming and expensive steps of sample collection, data collection and identification, and vouchering.

Finally and most importantly, biobanks offer barcoding projects the possibility to adequately voucher their molecular samples and to warrant reproducibility of results.

Researchers involved in barcoding projects should make sure their samples are properly vouchered – morphologically AND molecularly. They can do this by depositing their samples at a dedicated natural history collection. Increasingly, these repositories are establishing biobanks / DNA banks / tissue banks for curated long-term, ultra cold conservation of molecular samples, are adopting standard operating procedures and making their samples available online e.g. through biobank networks like the DNA Bank Network (http://www.dnabank-network.org/) or soon also the Global Genome Biodiversity Network (http://ggbn.org/). Those museums and natural history collections that implement these features and commit themselves to provide the community with proper biobanks (although maybe called differently) offer a very efficient and elegant way to both draw on and to deposit morphological-molecular ‘tandem’ samples. Often underappreciated by public and policy-makers (Suarez and Tsutsui 2004), natural history collections holding and curating specimen vouchers and/or cross-referenced molecular vouchers and their data play a “major role in organizing systematic knowledge in the molecular age” (Whitfield and Cameron 1994).

Although it has been pointed out before (e.g. Hafner 1994), the importance of vouchering molecular samples is not yet fully apprehended in the scientific community (perhaps because of the way taxonomy has been traditionally carried out).

We would like to encourage authors, editors and reviewers of scientific papers to give also molecular vouchers the attention they deserve.

Vouchers – morphological and molecular alike – not only form the connection between study data and taxonomic identification. They are much more: vouchers link the data collected in individual studies with the immense wealth of data that can still be (or already have been) collected through the vouchers: repetitively or in an additive manner. Put short, vouchers link individual studies with other studies and inferences, past or future.

It becomes obvious that it is only through adequate vouchering that we can make organismic biology meaningful, warranting reproducibility and embedding our research into existing and emerging knowledge.

In a laudable approach to increasing semantic accuracy regarding the voucher concept, Pleijel et al. (2008) suggest a terminology for those specimen vouchers used to produce molecular (sub-)samples. These are coined ‘genophores’ (although of course molecular samples lend themselves to more than genetic analysis), and for mnemonic ease follow the taxonomic nomenclatorial codes in style:

a *hologenophore* is the specimen voucher from which the molecular sample is directly derived, an *isogenophore* is a different specimen with a clonal relationship to the study organism, while a *progenophore* represents a voucher that is linked to the specimen sampled for molecular analysis by a parent-descendant or sibling relationship. A *paragenophore* is a putatively conspecific specimen voucher collected together with the ‘molecular’ specimen. The same applies to the *syngenophore*, except that it is collected at another place or time.

These genealogy-based distinctions made by Pleijel et al. (2008) are helpful for categorizing a specimen voucher in its relation to a molecular voucher and we endorse their use in this context. The function/purpose or the nature of vouchers was deliberately not addressed by Pleijel and colleagues. However, especially in the context of molecular samples, we perceive the necessity to do so, as varying uses of terms can be observed (e.g. “DNA voucher” used synonymously for the DNA source or for the isolated DNA). Different use of terms makes it difficult to extract data from biological collection databases or from the literature in a semantically meaningful way. Therefore, in the following we propose some voucher, sample and repository definitions, with special focus on a molecular context.

- *specimen voucher*: a specimen serving as the basis for taxonomic identification and possibly also for other queries. A specimen voucher is often, but not necessarily a whole organism, or part of it (it can be a trace or ichnofossil, scats, eggs, images, etc.).
  Narrower terms: morphological voucher, acoustic voucher, e-voucher, etc.
  - *morphological voucher*: a specimen that allows the inspection of morphological characters.
  - *e-voucher*: digital objects that serve as vouchers (morphological, acoustic, etc.), e.g. sound recordings, audiovisual material, images, etc.
- *molecular voucher*: a sample that is deliberately preserved and curated in a way that will conserve its molecular properties for analysis. A molecular voucher should always be linked to a specimen voucher (which sometimes can be the same object if sufficient characters remain, see tissue voucher).
  Narrower terms: biobank voucher, DNA voucher, tissue voucher, RNA voucher, protein voucher, genomic sample, etc.
  - *tissue voucher*: tissue subsampled from a specimen – or the entire specimen –, preserved (usu. frozen) to keep its molecular properties (either fixed tissue or viable cells) for future analysis
  - *DNA voucher*: the isolated and preserved, frozen or dried (usu. genomic) DNA. As a derived sample, a DNA voucher should not – if anyhow possible – function as specimen voucher.
  - *biobank voucher*: any molecular voucher curated in a biobank. A biobank voucher is a *biobank sample* that links to other physical objects or data (other than their metadata), i.e. most biobank samples are (biobank) vouchers, as they usually link to a separate specimen voucher
  - *genomic sample*: preserved sample containing (isolated or as a constituent) a high percentage of an organism’s genome in widely unfragmented form
- *biobank*: a curated collection/repository of biological materials that warrants long-term integrity at molecular level, authenticity, availability and rights management of its samples by adhering to standard operating procedures (SOPs).
  Narrower terms: DNA bank, tissue bank, biodiversity biobank, etc.
  - *biodiversity biobank*: term currently used to refer to a biobank holding non-human samples
  - *genomic collection*: a molecular collection holding genomic samples
